# Goreisan as a successful adjuvant therapy of heart failure with preserved ejection fraction and advanced chronic kidney disease: a case report

**DOI:** 10.1093/omcr/omad116

**Published:** 2023-10-23

**Authors:** Ryuta Sugihara, Tatsuro Hashimura, Yasushi Sakata

**Affiliations:** Department of Cardiovascular Medicine, Kawachi General Hospital, 1-31 Yokomakura, Higashiosaka, Osaka 578-0954, Japan; Department of Cardiovascular Medicine, Osaka University Graduate School of Medicine, 2-2 Yamadaoka, Suita, Osaka 565-0871, Japan; Department of Cardiovascular Medicine, Kawachi General Hospital, 1-31 Yokomakura, Higashiosaka, Osaka 578-0954, Japan; Department of Cardiovascular Medicine, Osaka University Graduate School of Medicine, 2-2 Yamadaoka, Suita, Osaka 565-0871, Japan

## Abstract

Atrial functional mitral and tricuspid regurgitation due to atrial fibrillation (AF) are common causes of heart failure with preserved ejection fraction, but standard treatment with conventional diuretics can often lead to renal dysfunction. Kampo Goreisan, a traditional Eastern-Asian herbal medicine that regulates body water balance via the aquaporin-incorporated water reabsorption system can be used as an alternative therapy without causing renal burden. In this report, we describe a case of successful treatment with Goreisan of heart failure with preserved ejection fraction (HFpEF) due to atrial functional mitral and tricuspid regurgitation (AFMR/TR) receiving guideline-directed medical-therapy. Goreisan could afford amelioration of regurgitation and improvement bilateral systolic ventricular function without renal dysfunction. Thus, Goreisan may be a promising therapeutic option for patients refractory to conventional diuretics.

## INTRODUCTION

Atrial fibrillation (AF) can result in functional regurgitation of atrioventricular valves in patients with structurally normal valves, a condition referred to as atrial functional mitral regurgitation and tricuspid regurgitation (AFMR/TR). This can subsequently lead to the development of heart failure with preserved ejection fraction (HFpEF) [1]. Although invasive therapies such as surgical or percutaneous repair, and mechanical circulatory devices are available, medical management is the primary treatment approach for AFMR/TR according to current guidelines [2].

Goreisan, a herbal formula containing five ingredients, has been traditionally used as an aquaretic agent to treat fluid regulation disorders in Japan (Goreisan), China (Wulingsan), and Korea (Oryeongsan) [3]. According to recent study, Goreisan has been shown to inhibit the water channel aquaporin, making it a potential treatment option for fluid retention [4].

We report a case of an elderly male patient with HFpEF due to AFMR/TR and chronic kidney disease who responded well to the water-eliminating agent, Goreisan. Goreisan may have potential as a novel therapy for fluid retention in elderly patients.

## CASE REPORT

An 84-year-old male patient presented to our hospital due to a progressive worsening of dyspnoea (New York Heart functional classification; NYHA class III) and pitting leg oedema over a period of three months. His medical history included hypertension, dyslipidaemia, and allergic rhinitis. The patient had three admissions for acute decompensated heart failure due to permanent AF. During initial hospitalization at the age of 73, he was administered a low dose of azosemide (30 mg/day) and effectively managed. However, after discharge, there was a gradual progression of atrial enlargement, leading to deteriorating regurgitation of the mitral and tricuspid valves over a 3-year period. To address this deterioration, the dosage of azosemide was increased to the maximum recommended level in Japan (60 mg/day) at the age of 76. Mineralocorticoid receptor antagonist (spironolactone 25 mg/day) was discontinued due to the occurrence of gynecomastia and hyperkalaemia. Subsequently, furosemide was introduced at the age of 77 and escalated to a dosage of 60 mg/day at the age of 79. Despite these interventions, the patient’s condition continued to deteriorate for years, resulting in a second hospitalization at the age of 79. It was during this period that renal dysfunction emerged (chronic kidney disease stage G4 to 3a), prompting the initiation of treatment with tolvaptan (7.5 mg/day). Unfortunately, his condition deteriorated over a three-year period, necessitating a stepwise titration of tolvaptan to the maximum recommended daily dosage of 15 mg during his third hospitalization at the age of 82. Additionally, an angiotensin II receptor antagonist (candesartan 4 mg/day) was introduced with the expectation of its cardioprotective effects; however, it was discontinued due to the development of vascular oedema. Over the course of two years following the last hospitalization, his renal function further deteriorated (chronic kidney disease stage G3b to G4). Consequently, he developed exertional dyspnoea (NYHA class III), bilateral pitting oedema of lower extremities, and experienced a weight gain of three kilograms (from 61 kg to 64 kg) over the last three months. Valve repair was not an option as the patient declined invasive treatments.

**Figure 1:**
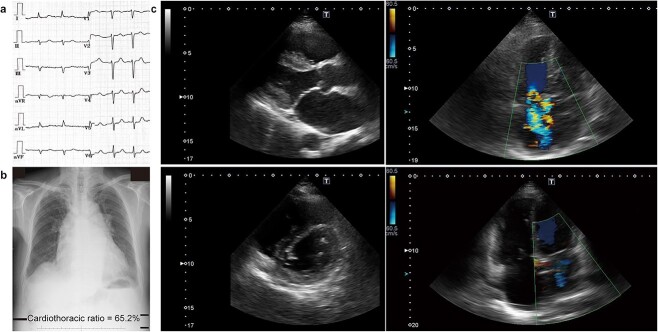
**Examinations before Goreisan treatment.** (**a**) Electrocardiogram shows atrial fibrillation with left anterior hemiblock. (**b**) Chest radiograph shows mild bilateral pulmonary congestion, moderate pleural effusion, and enlargement of the right ventricle and bilateral atrium. (**c**) Echocardiography shows dilation of left atrium (upper left panel), small diameter and D-shape of the left ventricle (lower left panel), severe tricuspid regurgitation (upper right panel), and moderate mitral regurgitation (lower right panel).

The patient’s blood test results revealed elevated levels of serum creatinine and brain natriuretic peptide (BNP) levels (60-90 ng/L). Electrocardiogram showed atrial fibrillation with left anterior hemiblock (Figure 1a). Chest radiography revealed mild bilateral pulmonary congestion, moderate pleural effusion, and enlargement of his heart (Figure 1b). Echocardiography indicated moderate mitral regurgitation and severe tricuspid regurgitation with pulmonary hypertension, as well as dilation of the right ventricle and bilateral atriums. The left ventricle had a D-shape with mild systolic dysfunction. (Figure 1c and Table 1).

**Table 1:** Changes in echocardiographic parameters following goreisan treatment

	Pre-goreisan	Post-goreisan
IVCd, maximum/minimum (mm)	28/24	29/18
RA short diameter (mm)	67	55
RA long diameter (mm)	57	59
RVDd (mm)	48	35
RVOTd (mm)	37	32
TAPSE (cm)	18	27
Tricuspid regurgitation	Severe	Severe
TRPG (mmHg)	31	22
PRedPG (mmHg)	7	0
LA volume index (ml/m^2^)	54	41
Mitral regurgitation	Moderate	Mild
LVOT-VTI (cm)	13	15.5
LV-SV (ml)	46	72
Ejection fraction (%)	62	69

IVCd; diamter of inferior vena cava, RA; right atrium, RVDd; right ventricular end-diastolic diameter, RVOTd; right ventricular outflow tract diamter, TAPSE; tricuspid annular plane systolic excursion, TRPG; peak systolic transtricuspid pressure gradient, PRedPG; peak early diastolic transpulmonary valve pressure gradient, LA; left atrium, LVOT-VTI; left ventricular outflow tract velocity time integral, LV-SV; left ventricular systolic volume.

**Figure 2:**
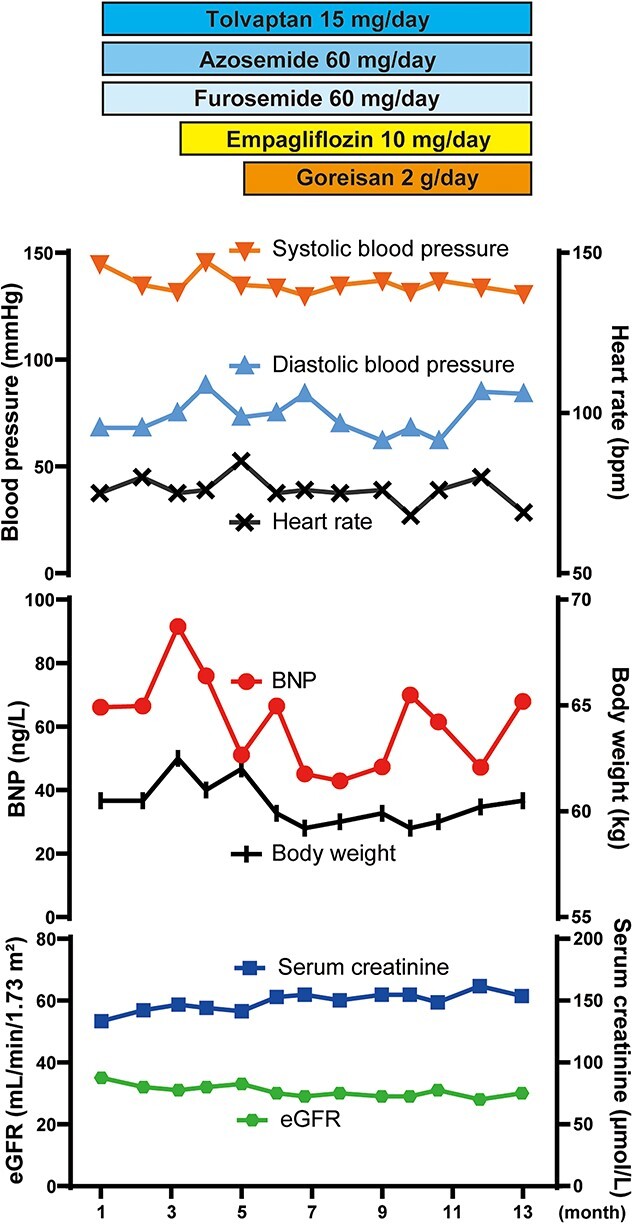
**Clinical time course of this patient.** Goreisan was administered in addition to tolvaptan, azosemide, and furosemide. BNP; B-type natriuretic peptide, eGFR; estimated glomerular filtration rate.

**Figure 3:**
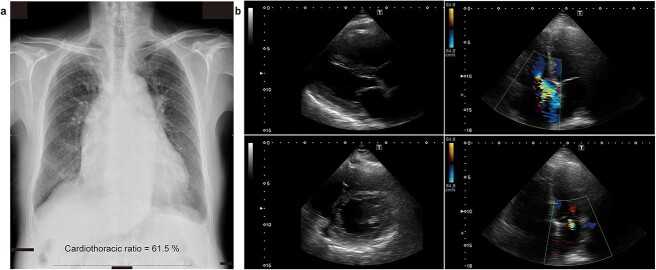
**Examinations after Goreisan treatment.** (**a**) Chest radiograph shows significant reduction in cardiac enlargement and pleural effusion. (**b**) Echocardiography shows decreased left atrial enlargement (upper left panel), disappearance of left ventricular D-shape (lower left panel), residual severe tricuspid regurgitation (upper right panel), and reduction in mitral regurgitation (lower right panel).

Despite the use of empagliflozin (10 mg/day) for two months, the patient’s symptoms and cardiac hemodynamic did not improve. This patient declined the use of an angiotensin receptor-neprilysin inhibitor or a mineralocorticoid receptor antagonist due to concerns about potential side effects. Goreisan was added as a subsequent treatment (1 packet contains 1 g of extract, taken twice a day within 30 minutes before breakfast and dinner) (Figure 2). Two months after initiating Goreisan treatment, the patient exhibited marked symptomatic improvement from NYHA class III to class II, despite a slight decrease in serum BNP levels (40-60 ng/L) and body weight (59-60 kg). There were tiny signs suggesting dehydration in blood examination (Table 2). Notably, serum creatinine, estimated glomerular filtration rate (eGFR, chronic kidney disease stage G3b), blood pressure and pulse rate remained stable (Figure 2). Moreover, a fluid retention effect was observed, as evidenced by the decrease in urine osmolality and increase in serum osmolality (Table 2). Chest radiography revealed a marked reduction in cardiac enlargement (Figure 3a). Surprisingly, echocardiography demonstrated improvements in right atrial pressure, pulmonary artery pressure, bilateral chambers volume and systolic functions, as indicated by tricuspid annular plane systolic excursion (TAPSE) and Left Ventricular Outflow Tract Velocity Time Integral (LVOT-VTI) (Table 1 and Figure 3b). After a 10-month follow-up period following the introduction of Goreisan, the patient’s symptoms have remained stable at NYHA class II, with no observed increase in BNP levels (40-60 ng/L). Furthermore, there has been no evidence of further deterioration in renal function and body weight. He is now undergoing monthly follow-up at our hospital.

**Table 2:** Changes in urinary and blood examinations following goreisan treatment

	Pre-goreisan	Post-goreisan
Osmolarity (mOsm/kg)		
Urine	305	286
Serum	294	304
Serum sodium (mmol/L)	141.0	144.0
Blood urea nitrogen (mmol/L)	7.00	9.21
Blood grucose (mmol/L)	5.33	6.88
Serum Creatinine(μmol/L)	142.3	153.8
Serum potassium (mmol/L)	4.3	3.8
Hematocrit (/L)	0.362	0.342
Serum albumin (g/L)	44.0	42.0
Hemoglobin (g/L)	112.0	106.0

## DISCUSSION

**Figure 4:**
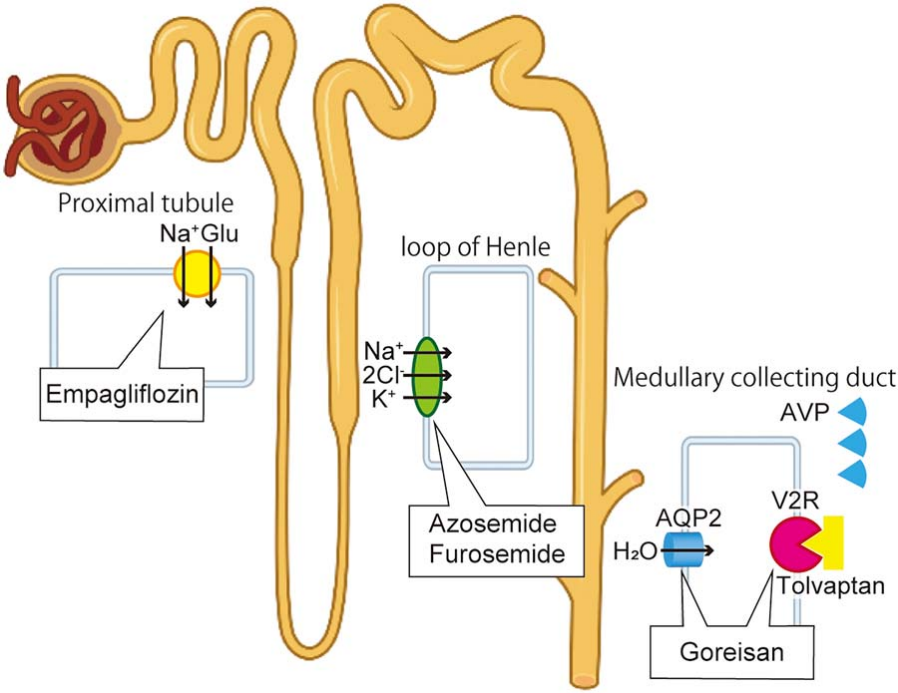
**Schematic illustration of diuretic-related drugs used in this patient.** In the proximal tubule, empagliflozin inhibits the sodium (Na^+^)-glucose (Glu) co-transporter 2 to reduce glucose reabsorption, resulting in increased urinary sodium and glucose excretion. In the thick ascending limb of Henle’s loop, azosemide and furosemide inhibit the sodium-potassium (K^+^)-chloride (Cl^−^) co-transporter, which can lead to significant diuresis. In the medullary collecting duct, tolvaptan blocks the V2 receptor (V2R) and reduces the effect of arginine/vasopressin (AVP) in the renal water reabsorption channel, aquaporin-2 (AQP2). Goreisan can inhibit the expression level of both V2R and AQP2 in the medullary collecting duct. This illustration of nephron was adapted from a design by BioRender.com.

Goreisan is known for its aquaretic properties, which effectively mitigate extravascular fluid leakage and provide therapeutic benefits in the management of oedematous conditions. In cerebral infarction, its effects have been attributed to the inhibition of aquaporin 4 channel expression, leading to amelioration of cerebral oedema [5]. Additionally, in cases of diarrhoea, Goreisan has been found to increase the expression level of aquaporin 3 in intestine, and ameliorate symptoms [6]. Moreover, in heart failure and chronic kidney disease, Goreisan has demonstrated the ability to suppress the expression level of aquaporin 2 in the kidney medulla, elevate atrial natriuretic peptide, and effectively control urinary reabsorption [7, 8]. Although we did not directly measure the expression level of aquaporin 2, it is plausible to consider that a similar mechanism could have played a role in our patient (Figure 4).

The water-regulating effects of Goreisan have the potential to reduce the reliance on conventional loop diuretics and aid in preserving renal function, which were associated with poor prognosis in HFpEF patients [9]. Moreover, Goreisan has pleiotropic effects in cardiomyocytes where aquaporin 1, 4, and 7 are expressed, as it has been shown to suppress the upregulation of aquaporin 4 gene expression [5, 10]. Goreisan may directly protect cardiomyocytes, potentially improving bilateral ventricular function in our patient. In addition, the administration of an angiotensin II receptor antagonist or a mineralocorticoid receptor blocker was not continued due to side effects in this case. It seems that the progression of renal dysfunction, alongside impairment of the renin-angiotensin-aldosterone system and diminished potassium excretion played contributory roles. Goreisan has the ability to regulate renin-angiotensin-aldosterone system, and provide a direct cardioprotective effect [7]. Consequently, it is conceivable that Goreisan may assume a heightened therapeutic significance in cases with heart failure who these agents are contraindicated due to renal dysfunction.

## CONCLUSION

As we have observed, Goreisan is a promising therapeutic option for heart failure with preserved ejection fraction due to atrial functional mitral and tricuspid regurgitation. In addition, it has a reno-protective effect by improving and stabilizing renal function in advanced CKD. Further studies are needed to substantiate these findings.

## Data Availability

Data sharing is not applicable to this article as no data sets were generated or analyzed in this article.
